# Metabolic Crosstalk in Multimorbidity: Identifying Compensatory Effects Among Diabetes, Hypertension, and Dyslipidemia

**DOI:** 10.1210/jendso/bvae152

**Published:** 2024-09-11

**Authors:** Erica Pitti, Domitilla Vanni, Nicola Viceconte, Angelo Lembo, Gaetano Tanzilli, Valeria Raparelli, Greta Petrella, Daniel O Cicero

**Affiliations:** Department of Chemical Science and Technology, University of Rome “Tor Vergata,” 00133 Rome, Italy; Department of Chemical Science and Technology, University of Rome “Tor Vergata,” 00133 Rome, Italy; Department of Cardiovascular, Respiratory, Nephrological, Anesthesiologic and Geriatric Sciences, Sapienza University of Rome, Policlinic Umberto I, 00161 Rome, Italy; Department of Chemical Science and Technology, University of Rome “Tor Vergata,” 00133 Rome, Italy; Department of Cardiovascular, Respiratory, Nephrological, Anesthesiologic and Geriatric Sciences, Sapienza University of Rome, Policlinic Umberto I, 00161 Rome, Italy; Department of Translational and Precision Medicine, Sapienza University of Rome, 00185 Rome, Italy; Department of Chemical Science and Technology, University of Rome “Tor Vergata,” 00133 Rome, Italy; Department of Chemical Science and Technology, University of Rome “Tor Vergata,” 00133 Rome, Italy

**Keywords:** NMR, metabolomics, multimorbidity, metabolic interference among diabetes, hypertension, dyslipidemia, biomarkers

## Abstract

**Context:**

Metabolomics is becoming increasingly popular for detecting markers that indicate the presence of a specific disease. However, it is usually applied to studying individual ailments, yielding results that may not be directly relevant to people with multiple health conditions.

**Objective:**

Our study proposes a different approach to explore metabolic crosstalk between various disease states.

**Design, Setting, and Patients:**

We conducted a study on subjects at medium to high risk of developing coronary artery disease. We measured the plasma levels of 83 metabolites using nuclear magnetic resonance and analyzed the connections between these metabolites and various risk factors such as diabetes, hypertension, and dyslipidemia. Linear regression and multivariate analysis were combined for this purpose.

**Results:**

Inspection of the metabolic maps created by our analysis helped us efficiently compare profiles. In this way, it was possible to discover opposing metabolic features among single conditions and their combination. Furthermore, we found compensating metabolic effects between diabetes, hypertension, and dyslipidemia involving mainly ketone body metabolism and fatty acid β-oxidation.

**Conclusion:**

Our study introduces a novel approach to investigating how metabolism reacts to the simultaneous presence of multiple health conditions. This has allowed the detection of potential compensatory effects between diabetes, hypertension, and dyslipidemia, highlighting the complexity of metabolic crosstalk in patients with comorbidities. A better understanding of metabolic crosstalk like this could aid in developing focused treatments, resulting in improved therapeutic results.

Metabolites found in human biofluids can serve as biomarkers that connect genetics, environment, and phenotype for clinical purposes such as diagnosis, prognosis, and disease classification [1]. These metabolites create a distinct profile that reflects the individual's health or disease status and offers insights into various physiological processes. Understanding the changes in metabolic pathways and identifying small molecule metabolites is crucial for comprehending the pathophysiology of a disease and identifying potential therapeutic targets [2].

Despite the promising results achieved in laboratory studies, there is a significant challenge in making these findings useful for clinical and industrial applications. In the future, research should address the main challenges: limited sample sizes, validating biomarkers in clinical settings, and real-time analysis of metabolites [3]. Moreover, most biomarker research is still based on a single-disease model, which may not be suitable for patients with overlapping health issues. However, patients with multiple conditions are more common than not in primary care. The prevalence of multimorbidity among adults is 37.2% globally, with South America having the highest percentage (45.7%), followed by North America (43.1%) and Asia (35%) [4]. Clinical trials and studies aimed at finding biomarkers tend to focus on efficacy rather than effectiveness, which means that patients with multiple conditions are often excluded. This exclusion can compromise the findings’ relevance when dealing with patients with various conditions or multimorbidity [5].

Individuals who are likely to develop cardiovascular disease (CVD) are an appropriate group to study the comorbidity phenomenon. The prevention and treatment of associated risk factors through medication have increased the age at which people experience their first cardiovascular event [6]. Consequently, people are surviving for longer periods after the onset of CVD, which often results in additional comorbid conditions. On the other hand, different pathologies can increase the risk of developing CVD, leading to a higher number of patients with more than 1 condition.

Among patients with CVD, coronary artery disease (CAD) is the most common type, prevailing in 66% and 53% of males and females, respectively [7]. CAD is characterized by the narrowing or blockage of coronary arteries, leading to reduced blood flow to the heart muscle and potentially resulting in myocardial ischemia and potentially acute myocardial infarction, heart attacks, angina, or heart failure. Various modifiable and nonmodifiable risk factors influence the pathogenesis of CAD. Modifiable risk factors include hypertension, dyslipidemia (Dy), diabetes, smoking, obesity, and a sedentary lifestyle [7]. Nonmodifiable factors include age, sex, and family history of cardiovascular disease. Hypertension often occurs with diabetes and cardiometabolic syndrome, which are characterized by metabolic abnormalities such as hyperglycemia, insulin resistance, Dy, and abdominal obesity [8]. Each condition introduces its own set of metabolic disturbances and biomarkers into the equation. Understanding how these metabolic derangements influence one another and contribute to the overall metabolic profile is a complex and relatively underexplored research area.

This research introduces a novel approach to investigating the interplay among metabolic markers when an individual suffers from more than 1 disease. Our findings reveal that specific metabolic imbalances caused by different health conditions can show opposite directions and may compensate partially, yielding a distinct biomarker signature. For this purpose, we examined the metabolic profile in the plasma of 306 patients at medium to high risk of developing CAD. Among these patients, 94 (31%) had diabetes, 241 (79%) had hypertension, 178 (58%) had Dy, and 10 (3%) had chronic kidney disease (CKD). These conditions were complexly intertwined, as only one-third of the patients had a single condition, and one-tenth had none. This clustering of diseases is likely due to shared risk factors such as aging, obesity, and smoking. Previous research has demonstrated that when 2 risk factors are present, the risk of CVD increases [9]. Patients with diabetes are 2 to 4 times more likely to develop CVD compared to those without diabetes [10]. Patients with cardiometabolic syndrome have a 5- to 9-fold increased risk of developing diabetes and a 2- to 4-fold increased risk of developing CVD [11]. However, limited evidence exists regarding the relationship between multiple chronic diseases and changes in circulating metabolite levels. Therefore, studying a diverse range of chronic diseases in a fixed population is necessary to understand metabolic responses and possible disease interactions.

Using a dataset comprising 83 distinct metabolites in plasma (including polar metabolites and lipid families), we combined multiple linear regression and multivariate analysis to examine the connections between these metabolites and different CAD risk factors, considered alone or in combination. The proposed approach provides a widely applicable framework for comparing conditions beyond binary classifications, enabling the detection of potential compensatory/additive metabolic effects in multimorbid states.

## Materials and Methods

### Plasma Sample Collection

A cohort of 306 clinical patients of the cardiological unit of Policlinico Umberto I of Rome were recruited for this study. All the studies on patients’ samples were approved by the Institutional Ethical Committee of the University of Rome “La Sapienza”—Azienda Policlinico Umberto I of Rome (study: RBSI14HNVT), and informed consent was obtained. All experimental procedures involving human biological material were conducted in accordance with approved guidelines. The study was conducted following the Declaration of Helsinki. An appropriate volume (2-3 mL) of venous plasma (Li-heparin) was collected. All samples were stored at −80 °C until analysis.

### Plasma Sample Preparation and Lipid Extraction

The plasma's lipid and polar fractions were extracted from a single sample aliquot. Deproteination of the plasma samples was performed by centrifugation with 3 kDa cut-off Amicon Ultra-0.5 centrifugal filter devices. The filters were previously washed 4 times to remove glycerol with 500 µL of distilled water by centrifugation at 13 800 g, 4 °C for 20 minutes. Then, 500 μL of each plasma sample was centrifuged at 13 800 g, 4 °C for 90 minutes, and the filtered volumes were collected. To each filtered sample, 100 μL of a buffer solution containing 250 mM phosphate buffer at pH 7.4, 1.05 mM sodium trimethylsilyl propanoate-d4 (TSP), 10% D2O, and 0.2% NaN3 were added. If the volume of filtered plasma was less than 350 μL, a measured amount of the filtrate was mixed with distilled water to make a total volume of 500 μL. This mixture was then transferred to a 5 mm nuclear magnetic resonance (NMR) tube for further analysis.

The concentrated portion on the top of the filter was processed to extract the lipid content from the plasma sample. To this aim, the filter device was turned upside down in a clean centrifuge tube and spun for 2 minutes at 1000 g to transfer the concentrated sample to the tube. Then, 250 μL of MeOH was added to the lipid-protein enriched portion of plasma, and the sample was stirred by vortex at 1800rpm for a few seconds and located in a thermomixer (25/28 °C) at maximum speed for 5 minutes. Then, 500 μL of chloroform was added, and the sample was stirred by vortex starting from 1800rpm to the maximum speed for a few seconds and then stirred by thermomixer (25/28 °C) at maximum speed for 15 minutes. Finally, 750 μL of 0.15 M NaCl aqueous solution was added to the organic mixture; the resulting biphasic mixture was stirred by vortex starting from 1800rpm to the maximum speed for a few seconds and then mixed by thermomixer (25/28 °C) at maximum speed for 5 minutes. The sample was centrifuged at 3300 g, 4 °C, for 10 minutes, and then 450 μL of the bottom organic phase was collected and dried under nitrogen flow at 37 °C. The dried lipidic extract was resuspended in 600 uL of CDCl3 containing 0.05% tetramethylsilane (TMS; internal standard) and transferred in a 5 mm NMR tube for the following acquisition.

### NMR Spectroscopy

^1^H-NMR experiments were acquired on a Bruker Avance 700 MHz equipped with an EasyJet autosampler, 5 mm inverse broadband probe, and Z-gradients for the polar fraction. For each plasma sample, a ^1^H NMR spectrum was acquired at 25 °C using the noesypr19d pulse sequence with a mixing time of 100 ms, spectral width of 12 ppm, acquisition time of 2 seconds, and relaxation delay of 3 seconds. If the sample contained 350 μL of filtered plasma, 256 scans were acquired; otherwise, scans were increased to maintain a good signal to noise ratio. Before each acquisition, ^1^H pulse length and water suppression were checked to verify the instrument's performance. Moreover, the ^1^H-^29^Si coupling peaks of the TSP (or TMS for lipid extracts) should be resolved. NMR free induction decay (FIDs) were imported in the Chenomx NMR suite (version 8.1). This software was used to identify and quantify plasma metabolites in each spectrum. Chenomx specializes in mixture analysis for applications in life sciences such as metabolomics, food/nutrition, and cell culture research. It works with comprehensive Spectral Reference Libraries to both identify and measure concentrations of compounds visible in the NMR spectra, all in 1 integrated workflow. Once imported, NMR FIDs were processed with the Processor function applying a manual phase and baseline correction, a line broadening of 0.5 Hz, an automatic shim correction, and the TSP calibration (setting up the final concentration value of 0.21 mM). Each spectrum was manually assigned through the NMR Suite Profiler, and 56 metabolites were identified and quantified.

Lipid extracts suspended in 600 μL of chloroform-d containing 0.05% TMS. We employed a combination of ^1^H and ^1^H-^1^H TOCSY NMR spectra for lipidome characterization, which yielded the most information-rich yet time-efficient data. Data reduction techniques were implemented to eliminate redundant variables representing identical lipid species. Spectra were acquired on Bruker Avance 700 MHz spectrometer (Billerica, MA, USA) equipped with a Triple resonance TXI probe and a Sample Xpress Lite autosampler. ^1^H-NMR spectra were acquired at 25 °C with a spectral width of 20 ppm, 64 scans, 4 dummy scans, and a total acquisition time of 5 minutes. For each sample, a ^1^H-^1^H TOCSY NMR spectrum was also acquired at 25 °C with a spectral width of 8 ppm in F1 and 12 ppm in F2, mixing time of 80 ms, 4096 points of FID in F2, and 128 points in F1, 8 scans, 8 dummy scans, and total acquisition time of 40 minutes. The spectra were processed using Topspin 3.6.2. ^1^H chemical shifts were referenced to TMS at 0 ppm.

We analyzed the lipid compositions of 9 human plasma samples by pooling extracts to address individual differences and increase sample concentration. In addition to a 1D proton experiment, we performed 2D experiments to characterize the lipid spectrum better and assess their value for lipidomics studies. Spectral assignments were based on resonance values from previous studies [12].

In the initial 1D experiment, a single lipid extract was analyzed, taking 5 minutes. The spectrum was divided into regions representing different lipid classes. However, signal overlap made it challenging to identify some fatty acids. The ^1^H-^1^H TOCSY experiment was also chosen for better data quality, reducing the acquisition time from 2 to 40 minutes while maintaining peak resolution. This experiment enabled discrimination of previously overlapped species, including monounsaturated fatty acids (FAs), ω-3 FAs, and phosphoethanolamine-containing lipids (PE) and sphingomyelin (SM).

Following the integration and comparison process, a dataset of 63 variables was obtained and refined to 15 distinct variables after a clustering step. These variables represent various quantifiable lipid species, including cholesterol, phospholipids, glycerolipids, sphingolipids, and FAs.

### Data Analysis: Multiple Linear Regression and Multivariate Statistical Analysis

Before statistical analysis, the metabolite concentrations and lipid species’ relative intensities were normalized using probabilistic quotient normalization [13]. The missing values are randomly distributed, accounting for 1.4% of the data. The multiple linear regression model was performed in R version 4.1. In the data preparation phase, missing values in the dependent variable were addressed using listwise deletion (complete case analysis). This means that missing values were left as blanks. This ensures that the original structure and integrity of the data are preserved, avoiding the introduction of bias through imputation. In addition, no additional assumptions about the data's distribution or the missingness mechanism were made. The independent variables, primarily clinical and demographic conditions, were collected from a cohort of 306 patients and imported into R for further analysis. The dependent variable set consists of the metabolite concentrations and the lipid relative intensities.

To calculate the metabolic profile associated with single conditions, each concentration of the 83 metabolites or lipid families, *M_i_*, was linearly correlated with the nine conditions *C_j_*: age, sex (1 representing female), diabetes, hypertension, Dy, CKD, and the use of antiplatelet drugs for preventing blood clot formation (Apd), statins for dyslipidemia (Std), or hypertension (Hyd), according to Eq. 1.


(1) $$M_{i}=\overset{9}{\underset{\;j=1}{\sum }}\;\beta_{ij}C_{j}$$

For each regression coefficient, $\beta_{ij}$, in Eq. 1, the respective *P*-value, *p_ij_* representing the significance of the predictors (the 9 conditions) in explaining the variation in the response (the metabolite levels), was calculated. They have been log-transformed and multiplied by the sign of the coefficient, *s_ij_*, to yield the *v_ij_* elements of an 83 × 9 matrix:


(2) $$v_{ij}=-s_{ij}\log_{10}\;(\;p_{ij})\;$$

This matrix was transposed and used as input for the subsequent multivariate analysis.

An analogous procedure was followed to calculate the metabolic profile associated with multimorbid conditions. For example, in the case of diabetes/hypertension (DH), the conditions considered were DH, Dy, CKD, Apd, Std, and Hyd. The variable DH was constructed considering subjects with both conditions (DH = 1) and subjects free of both (DH = 0). The same procedure was adopted for diabetes/dyslipidemia (DDy), hypertension/dyslipidemia (HDy), and the 3 coexisting conditions (DHDy).

The principal component analysis (PCA) was conducted with SIMCA (version 17.0.1. Umetrics AB, Umea, Sweden). The *v_ij_* values were scaled using the unit variation formula. The biplot representation used to compare the different conditions is a scaled overlapping of the score and loading plot to simultaneously visualize the relative position of the observations and the variables. This plot shows similarities and differences between observations and allows us to understand the observations in terms of variables. Observations located near variables are high in these variables, while those located opposite are low. Observations close to the plot origin have average properties and are poorly described by these model components.

The regular loading values (p) represent the covariances between the X-variables and the score vectors (t). This means that the magnitude of each X-variable is reflected in the loading value. The loadings are scaled as correlations rather than covariances to remove this influence. After this rescaling, the loading values of all loading vectors range between −1 and +1. To enable the biplot, the score vectors must also be rescaled into the −1 and +1 numerical range. This is accomplished using a scaling factor based on a ratio of the sum of squares of the loadings to the sum of squares of the scores.

### Pathway Analysis

MetaboAnalyst's enrichment analysis [14] was used to identify overrepresented metabolic pathways or compounds from the list of metabolites associated with a given condition. This list was compared to the reference set embedded into the Small Molecule Pathway Database [15], containing 99 metabolite sets based on normal human metabolic pathways. The process involves metabolite identification, data mapping, and statistical enrichment analysis. MetaboAnalyst applies the hypergeometric test or Fisher's exact test to evaluate the overrepresented metabolites in pathways or sets, correcting *P*-values with the false discovery rate to control the risk of type I errors.

## Results

### Demographic and Clinical Profile of the Cohort

A total of 306 adult subjects, identified as intermediate-to-high-risk candidates for CAD, were enrolled in the present study. Our cohort consisted mostly of males and individuals aged 65 or older, representing the at-risk population for CAD [16]. For this study, we excluded those patients who presented type I diabetes and those with type II diabetes not treated with oral hypoglycemic drugs. The diagnosis of diabetes was confirmed in patients with glucose levels greater than or equal to 7.0 mmol/L (or 126 mg/dL) after at least 8 hours of no caloric intake [17]. Patients with systolic blood pressure of 140 mmHg or higher and diastolic blood pressure of 90 mmHg or higher were classified as having hypertension, according to the 2023 European guidelines [18]. No distinction was made between different grades of hypertension, and people with isolated systolic or diastolic hypertension were not included in the study. Dy was defined as having triglyceride levels above 240 mg/dL [19]. CKD was defined as kidney structure or function abnormalities lasting over 3 months [20].

Comprehensive demographic and health-related data are tabulated in Table 1. An analysis of this table reveals a prevalence of hypertension, followed by Dy and diabetes. Most subjects presenting these 3 conditions were males. Only a few present CKD were free of cardiovascular risk factors and were equally distributed between the 2 sexes. Among the participants, 76% were prescribed antihypertensive medications such as beta-blockers, angiotensin-converting enzyme inhibitors/angiotensin II receptor blockers, and calcium channel antagonists. Additionally, antiplatelet agents were given to 58% of the participants, mainly to reduce the risk of myocardial infarction. About 49% of the participants were under statin therapy, which aimed to lower low-density lipoprotein cholesterol levels.

**Table 1.** Baseline participant characteristics indicating the prevalence of the different CAD risk factors

	Diabetes	Hypertension	Dyslipidemia	CKD	Risk factors free	Total
n (%*^a^*)	94 (31)	240 (78)	177 (58)	10 (3)	31 (10)	306
Males (%*^b^*)	79 (84)	163 (68)	120 (68)	5 (50)	17 (55)	203 (66)
Age*^c^*	71 (52-85)	70 (34-88)	70 (36-88)	73 (45-81)	62 (48-85)	69 (34-88)
Treatments, n (%*^b^*)						
Statins	63 (67)	127 (53)	127 (72)	4 (40)	4 (13)	149 (49)
Antiplatelet	66 (70)	149 (62)	119 (67)	6 (60)	9 (29)	178 (58)
Antihypertensive	79 (84)	212 (88)	145 (82)	10 (100)	8 (26)	234 (76)

Abbreviations: CAD, coronary artery disease; CKD, chronic kidney disease.

^*a*^The percentage is calculated on the total of 306 participants.

^*b*^The percentage refers to the single condition.

^*c*^Average (range).

### Metabolomic Analysis via NMR Spectroscopy

We used NMR spectroscopy to analyze plasma samples’ metabolic profiles: a ^1^H-NMR experiment identified and quantified 56 metabolites (Table 2). Our optimized dual-phase approach separated lipid-rich lipoprotein fractions from the aqueous phase and extracted lipids via a modified Folch method, allowing simultaneous analysis of polar and lipid metabolites from a single sample, addressing resource and time constraints.

**Table 2.** List of metabolites and their respective abbreviations used in the text, whose circulating levels were obtained by NMR

Class	Metabolite	Abr.
Amino acids	alpha-aminobutyrate	aAB
	alanine	Ala
	arginine	Arg
	asparagine	Asn
	cystine	Cys
	histidine	His
	lysine	Lys
	phenylalanine	Phe
	proline	Pro
	pyroglutamate	Pyro
	taurine	Tau
	tyrosine	Tyr
BCAAs and metabolites	alpha-hydroxyisovalerate	aHIVA
	alpha-ketoisocaproate	aKIC
	3-hydroxyisobutyrate	bHIB
	beta-hydroxyisovalerate	bHIVA
	3-Methyl-2-oxovalerate	aKbIVA
	isoleucine	Ile
	leucine	Leu
	valine	Val
Dietary metabolites	dimethylamine	DMA
Energy	alpha-ketoglutarate	aKG
	citrate	Ctr
	creatine	Cr
	creatine phosphate	CrP
	glucose	Gluc
	lactate	Lact
	pyruvate	Pyr
	succinate	Suc
Fatty acids, triglycerides, and their metabolism	acetate	OAc
	betaine	Bet
	carnitine	Car
	glycerol	Glyc
	O-acetylcarnitine	OAcC
Glutamine and glutamate	glutamate	Glu
	glutamine	Gln
Glycine, serine, and methionine metabolism	choline	Ch
	formate	For
	glycine	Gly
	methionine	Met
	dimethylglycine	DMG
	sarcosine	Sar
	serine	Ser
	threonine	Thr
Ketone bodies and precursors	alpha-hydroxybutyrate	aHB
	beta-hydroxybutyrate	bHB
	acetoacetate	AcAc
	acetone	AC
Nucleotide metabolism	hypoxanthine	Hypox
Sulfur metabolism	dimethyl sulfone	DMS
Urea cycle and kidney function metabolites	citrulline	Cit
	creatinine	Crtn
	myo-inositol	mIno
	ornithine	Orn
	trimethylamine N-oxide	TMAO
	urea	Urea

Abbreviations: BCAA, branched-chain amino acids; NMR, nuclear magnetic resonance.

The lipidomic dataset encompassed 15 distinct variables, covering quantifiable lipid species, including free (FC), esterified (EC), and total cholesterol, phosphocholine-bearing lipids (PC) and SM, PE-bearing lipids and SM, glycerolipids, glycerophospholipids, sphingolipids, plasmalogens (PLS), total FAs, total unsaturated FAs, monounsaturated FAs and polyunsaturated FAs, their sum (unsaturated FAs), ω-3 FAs, linoleic acid, docosahexaenoic acid, and the sum of arachidonic acid and eicosapentaenoic (EPA) acids.

In addition to the individual variables, we also incorporated 6 metabolic ratios: those involving amino acids (AAs) (Phe/Tyr, Gln/Glu), the ketone bodies (KBs) acetoacetate and beta-hydroxybutyrate (AcAc/bHB), O-acetylcarnitine and carnitine (oAcC/Car), the 2 forms of cholesterol (EC/FC), and the ratio between FAs and ketone bodies (FA/KB). In addition, we considered 5 cumulative sums: total AAs, essential AAs (EAs), nonessential AAs, branched-chain AAs (BCAAs), and KBs. By amalgamating information from the polar and lipid sections, our final dataset comprised 83 variables, providing a comprehensive platform for our investigative pursuits.

### PCA of Multiple Linear Regression Results: Metabolic Relationship Among Individual Pathological Conditions

Our study employed a novel approach to compare metabolic profiles associated with diabetes, hypertension, Dy, sex, aging, and CKD. The traditional multivariate analysis in metabolomics considers subjects as observables and metabolite levels as variables. However, we opted for a different approach by calculating a PCA model where we treated conditions as observables and the product of the sign of the correlation and the -log10 transformed *P*-values obtained in multiple linear regression as variables. In this way, we considered 2 key components essential for interpreting the linear regression model. While the effect size (the coefficient value) can also be incorporated in future analyses, our current version deems the *P*-value and direction of the variation sufficient for extracting the most significant aspects of the relationship between the predictors (clinical conditions and treatments) and the response (metabolite levels). The metabolic profile associated with a given disease comprises the set of responses with which it is significantly associated.

Analysis of the metabolite levels performed by multiple linear regression produced a matrix of *P*-values with the associated signs of the regression coefficients for 83 metabolomic variables across 9 demographic, clinical, and treatment conditions. These predictors are age; sex; diabetes; hypertension; Dy; CKD; and the use of Apd, Std, and Hyd. This final matrix was then used to conduct a series of principal component analyses. The observables are the patients’ conditions, while the variables are the significance levels of the regression converted to a logarithmic scale and multiplied by the sign of the regression. In this way, the smaller the value *P*, the greater the value of its logarithm and, thus, the more pronounced its correlation to a given condition. On the other hand, the associated sign is used to distribute the relative position of the various observables. In a score plot of a PCA obtained with this matrix, the relative position of 1 observable with respect to another will thus be explained by its correlation (positive sign) and anticorrelation (negative sign) with a set of variables with high absolute values of -log(*P*-value).

**Figure 1.**
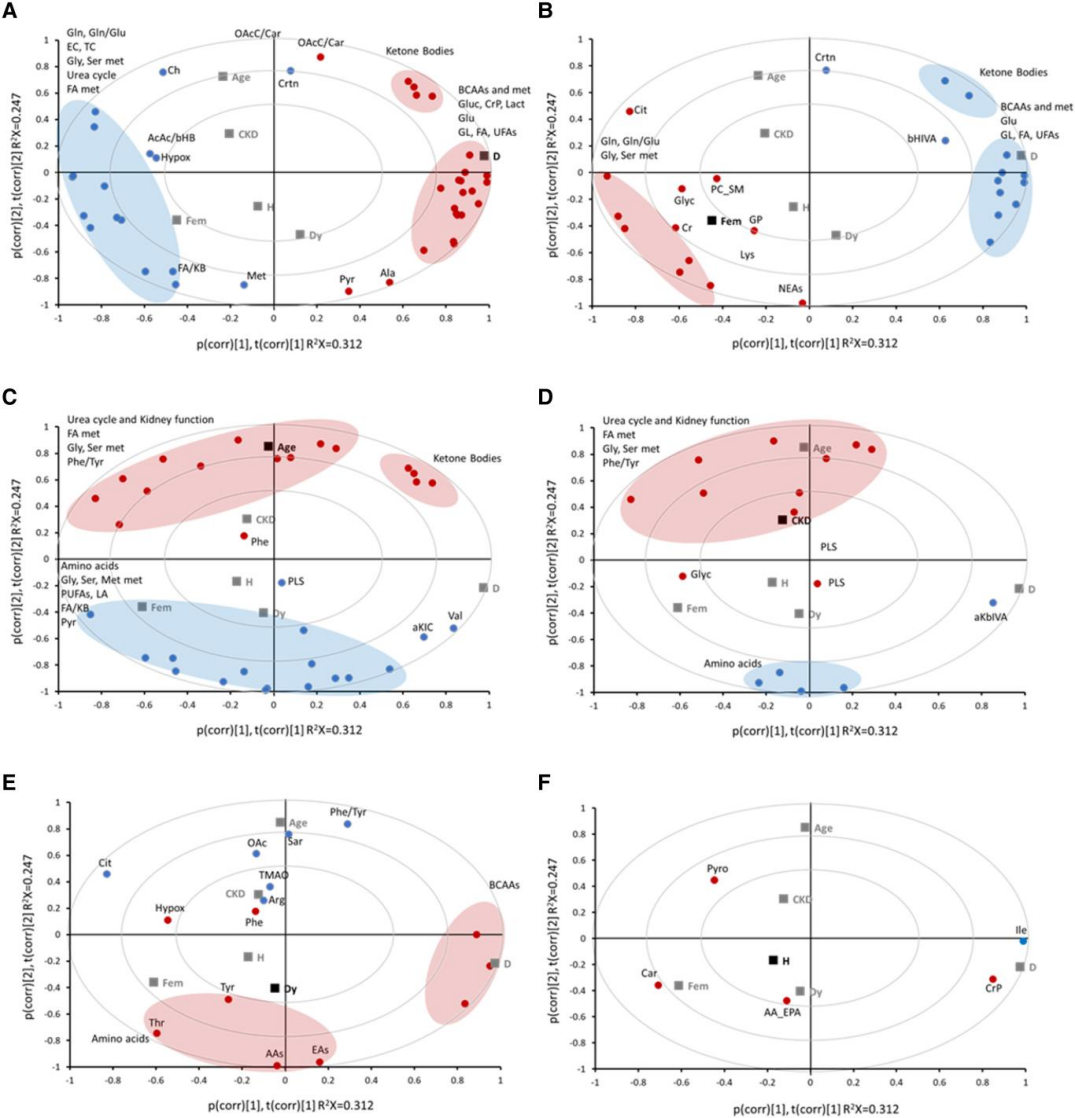
Biplots of the PCA analysis of the subject conditions using the -log(p)*sign as indicators of the metabolic profile, where p is the *P*-value of the linear regression for each condition with each metabolite level. Variables related to medication with antiplatelet, statins, or hypertension drugs were introduced to correct their effects, but they are not shown in the graph. The 6 independent variables are shown as black squares, and their position corresponds to the score plot of the PCA. The metabolite variables are arranged as in the loading plot. Each plot displays only metabolites significantly associated with diabetes (A), sex (B), age (C), CKD (D), dyslipidemia (E), and hypertension (F). Positive associations are indicated in red, and negative in blue. For the abbreviations used for polar metabolite names, please refer to Table 2. Abbreviations: CKD, chronic kidney disease; PCA, principal component analysis.

A series of biplots (Fig. 1A-1F) were generated, capturing the multidimensional relationships among metabolites and clinical conditions. Each indicates metabolites correlating with different diseases and distinguishes positive from negative correlations. The drug treatment variables were not incorporated into the PCA, although their inclusion helped to correct the correlations.

### Method Validation: Accurate and Selective Extraction of Biomarkers Across Single Conditions, Confirming Literature Data

PCA is usually performed on a correlation or covariance matrix. In our analysis, we entered the transformed regression coefficients into PCA and calculated a correlation matrix from the transformed coefficients obtained from the linear regression. Although it may seem redundant, this process allows for a straightforward interpretation of the results obtained from the biplots, as shown in Fig. 1. Additionally, since this statistical method has not been previously validated, we analyzed the biomarkers associated with the different conditions our method yielded and compared them with the vast literature data to validate the results.

The metabolic map depicted in Fig. 1A underscores the substantial impact of the diabetes variable on the first component of the PCA, positioning it on the far right of the biplot. This positioning arises from the positive associations of several metabolites and ratios, including circulating BCAAs and their metabolites; KBs; key energy metabolites like glucose, creatine phosphate, pyruvate, lactate; and AAs such as glutamate and alanine. Furthermore, we observed positive associations of elevated levels of FAs (total and unsaturated) and glycerolipids within the lipid composition with diabetes. Conversely, metabolite levels and ratios on the graph's opposite side show robust negative association with diabetes. These include metabolic pathways such as serine, glycine, and methionine (Gly, Met, Ser, Thr, Orn), the urea cycle (Cit and Orn), lipid metabolism (betaine, carnitine, choline), cholesterol (both esterified and total), Gln, and the Gln/Glu ratio. Additionally, ratios such as FA/KB, AcAc/bHB, and hypoxanthine also show negative associations with diabetes.

Interestingly, females’ metabolic profiles exhibit numerous contrasting associations relative to diabetes, as evident in the distinct correlation patterns depicted in Fig. 1B vs Fig. 1A. Within our cohort, BCAAs, KBs, glutamate, glycerolipids, and FAs display negative associations with females. In contrast, metabolites associated with glutamine, glycine, serine metabolism, and the Gln/Glu ratio display positive associations. It is worth noting that this profile has been adjusted for age and any concurrent illnesses or medications among the patients.

The variable “age’ emerges as the second most influential factor, impacting the second component, as indicated in Fig. 1C. Lipid-related metabolites (O-acetylcarnitine and the OAcC/Car ratio), KBs, urea cycle components, kidney function-related factors (creatinine, myo-inositol, urea), sarcosine, and AAs Phe and Cys, along with the Phe/Tyr ratio exhibits positive associations with aging. In contrast, total AAs, encompassing EAAs and nonessential AAs, particularly Ser, Met, His, and Thr, are negatively associated with age, as well as FA/KB ratio and other factors such as PLS, aKIC, and Val levels.

Upon comparing the biplots in Fig. 1D and 1C, it becomes evident that the metabolic profile associated with CKD bears striking similarities to aging. Predictably, metabolites linked to kidney function and the urea cycle, such as creatinine, citrulline, myo-inositol, dimethylglycine, and trimethylamine N-oxide (TMAO), positively correlate with CKD. Metabolites involved in FA metabolism, including choline and the OAcC/Car ratio, also showed a positive association, as did the Phe/Tyr ratio. Much like aging, CKD manifests negative correlations with the total content of AAs, EAAs, methionine, and asparagine.

Conversely, the metabolic profile associated with Dy presents distinct associations compared to aging and CKD (Fig. 1E). AAs and EAAs are positively associated, while sarcosine, citrulline, and TMAO exhibit negative associations. Furthermore, the Phe/Tyr ratio reveals a negative association. Positive associations are also observed for hypoxanthine, Phe, Tyr, and BCAAs, while OAc and Arg display negative associations.

Amino and EAAs positively associate with Dy, while sarcosine, citrulline, and TMAO exhibit negative associations (Fig. 1E). Furthermore, the Phe/Tyr ratio reveals a negative association. Positive associations are also observed for hypoxanthine, Phe, Tyr, and BCAAs, while OAc and Arg display negative associations.

In contrast, hypertension exhibits relatively few significant associations, as indicated in Fig. 1F, which visualizes the metabolites correlated with this condition. Positive associations are identified for carnitine, the sum of arachidonic acid and eicosapentaenoic acids, pyroglutamate, and creatine phosphate. Conversely, isoleucine demonstrates a negative correlation.

We compared our results with literature on known biomarkers associated with each condition. A detailed comparison is presented in the Discussion section. Our findings demonstrated a strong correlation between our data and published biomarkers, which serves as evidence of the validity of our approach.

### Metabolic Crosstalk in Multimorbid Conditions

**Figure 2.**
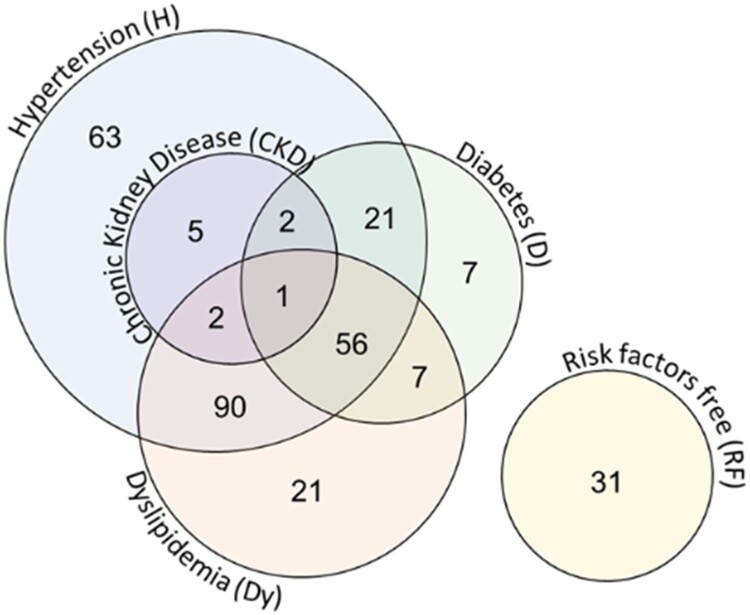
Venn's diagram of the distribution and the combination of the different conditions of the 306 patients.

Next, we investigated the metabolic crosstalk that appears when different illnesses are combined. Figure 2 displays the interwoven conditions within patient profiles. We used the same method for single conditions to calculate the association of mixed conditions (DH, DDy, HDy, and DHDy) with the different metabolite concentrations. For instance, when we calculated the associations for the DH state, we included patients with both diseases (DH = 1) or none (DH = 0) but not those with only 1 of them. We adjusted for age, sex, Dy, CKD, and the use of different drugs. We repeated this process for the other 3 mixed conditions. Table 3 shows the number of patients considered in these calculations and those used to determine the metabolic profile of the 3 single conditions.

**Table 3.** Number of patients included in the linear correlation regarding the single and comorbid states

Condition	No disease	Disease	Total
D	212	94	306
H	66	240	306
Dy	129	177	306
DH	52	80	132
DDy	99	64	153
HDy	38	149	187
DHDy	31	57	88

Abbreviations: D, diabetes; DDy, diabetes/dyslipidemia; DH, diabetes/hypertension; DHDy, diabetes, hypertension, and dyslipidemia; Dy, dyslipidemia; H, hypertension; HDy, hypertension and dyslipidemia.

**Figure 3.**
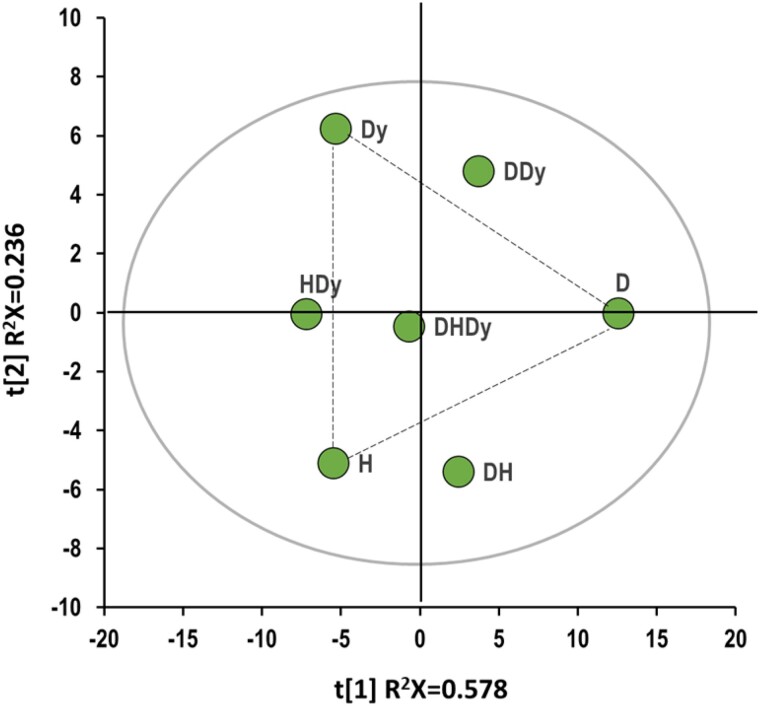
Principal component analysis of the conditions (D, H, Dy) and their combinations (DH, DHDy, DDy, HDy). The variables used to build this model are the product between the sign and the -log(p-value) obtained from the multiple linear regression. Abbreviations: D, diabetes; DDy, diabetes/dyslipidemia; DH, diabetes/hypertension; DHDy, diabetes, hypertension, and dyslipidemia; Dy, dyslipidemia; H, hypertension; HDy, hypertension and dyslipidemia.

We applied the same PCA model approach used for the analysis of single conditions to examine the correlated metabolic profiles associated with multiple health conditions (Fig. 3). The first component, which accounts for 58% of the total variance, effectively differentiated diabetes from Dy and hypertension. Simultaneously, the second principal component (24%) separated Dy from hypertension. The metabolic relationships among these conditions assumed a triangular configuration, with the single conditions occupying the vertices and the dual conditions aligned along the sides. The triple condition, denoted as DHDy, was positioned near the center of the triangle, indicating that combined conditions display association patterns that encompass traits from individual states but also possess distinctive characteristics. The relative positioning of HDy in relation to diabetes implies that specific metabolic associations between HDy and diabetes exhibit opposing trends. This phenomenon contributes to interferences in the metabolite concentrations and imparts a distinct profile to individuals with all 3 concurrent health conditions.

### Changes in the Metabolic Fingerprint of Diabetes in the Copresence of Dy and Hypertension

**Figure 4.**
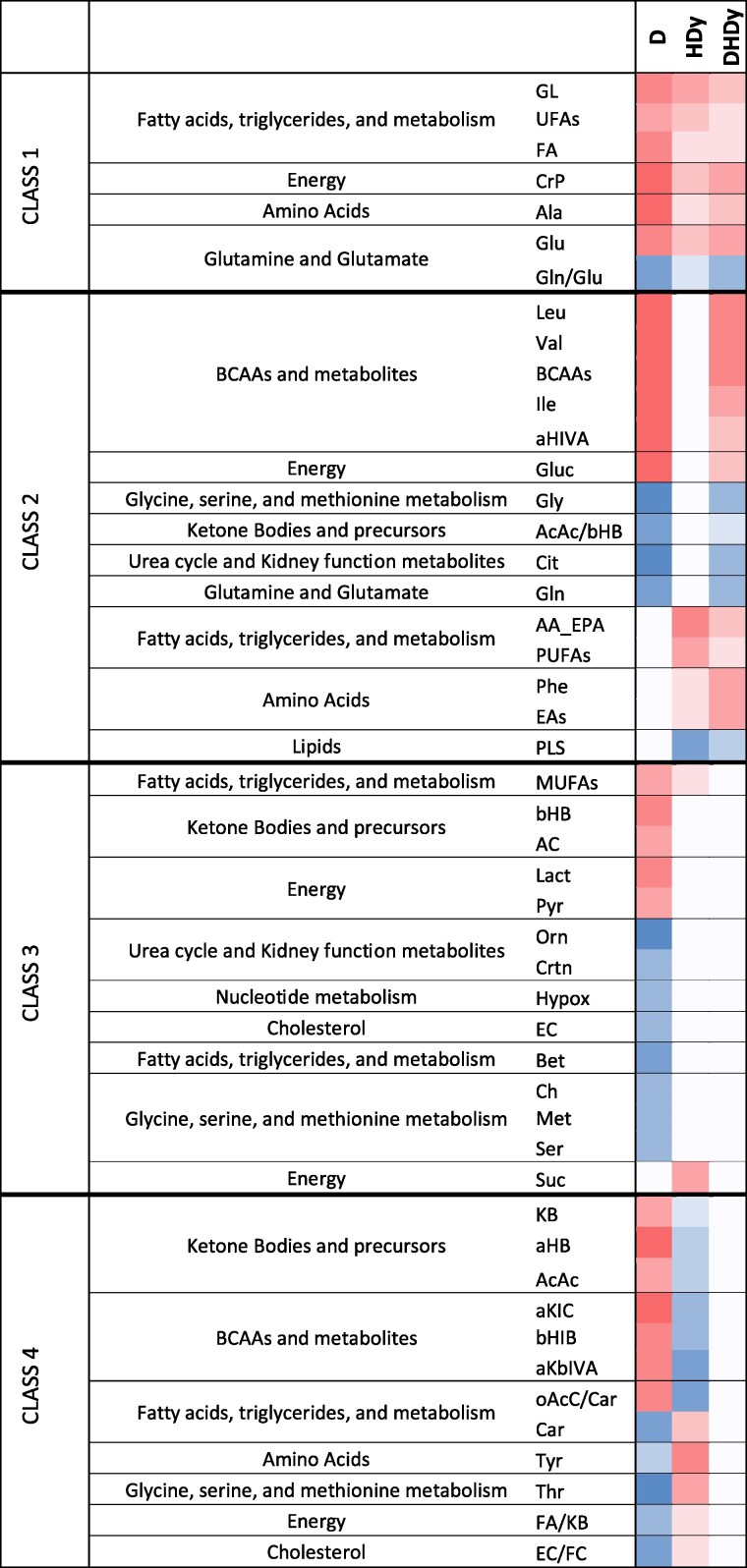
Metabolites with their respective metabolic class significantly associated (positively in red and negatively in blue) with D, HDy, and the triple condition (DHDy). The color gradient starts from a *P*-value of the correlation of 0.1 to a value <.001. Metabolites are divided into 4 main classes, as described in the text. Abbreviations: D, diabetes; DHDy, diabetes, hypertension, and dyslipidemia; HDy, hypertension and dyslipidemia.

We extended our exploration to investigate the combined impact of diabetes, hypertension, and Dy on the metabolic profile. We compared correlation profiles across 3 patient groups: those with diabetes only, those with all 3 conditions, and those with hypertension and Dy without diabetes, as summarized in Fig. 4. This selection was guided by the distinctive positioning of diabetes and HDy in the Fig. 3 score plot.

To gain insights into the influence of each condition on the metabolic profile, we categorized metabolite associations into 4 classes. The first class, detailed in the upper section of Fig. 4, encompasses metabolites exhibiting similar correlations in patients with diabetes alone and those with Dy and hypertension, thus maintaining these correlations in subjects presenting the 3 conditions. This class includes triglycerides, FAs, unsaturated FAs, creatinine phosphate, alanine, glutamate, and the Gln/Glu ratio.

The second category encompasses metabolites that correlate exclusively with either diabetes or HDy but uphold their associations with DHDy. This group includes BCAAs, glucose, glycine, the AcAc/Hb ratio, citrate, and glutamine, which correlate with diabetes, as well as the sum of arachidonic acid and eicosapentaenoic acids and polyunsaturated FAs, phenylalanine, EAAs, and PLS, which exclusively relate with HDy.

The third class comprises metabolites solely correlated with 1 condition. Metabolites in this class are KBs, lactate, and pyruvate, which positively correlate with diabetes but not with DHDy.

The fourth and final class is particularly interesting, as metabolites in this category display opposing correlations between diabetes and HDy, accounting for their contrasting positions in the Fig. 3 score plot. These metabolites include KBs and their precursors, along with metabolites related to BCAAs. In all instances, there is a positive correlation with diabetes and a negative correlation with HDy. The correlation signs of the OAcC/Car ratio, carnitine, tyrosine, and threonine levels are also reversed. The FA/KB and the EC/FC ratios complete the list of metabolites revealing negative interference between diabetes and HDy, contributing to their absence in the metabolic profile of DHDy.

**Figure 5.**
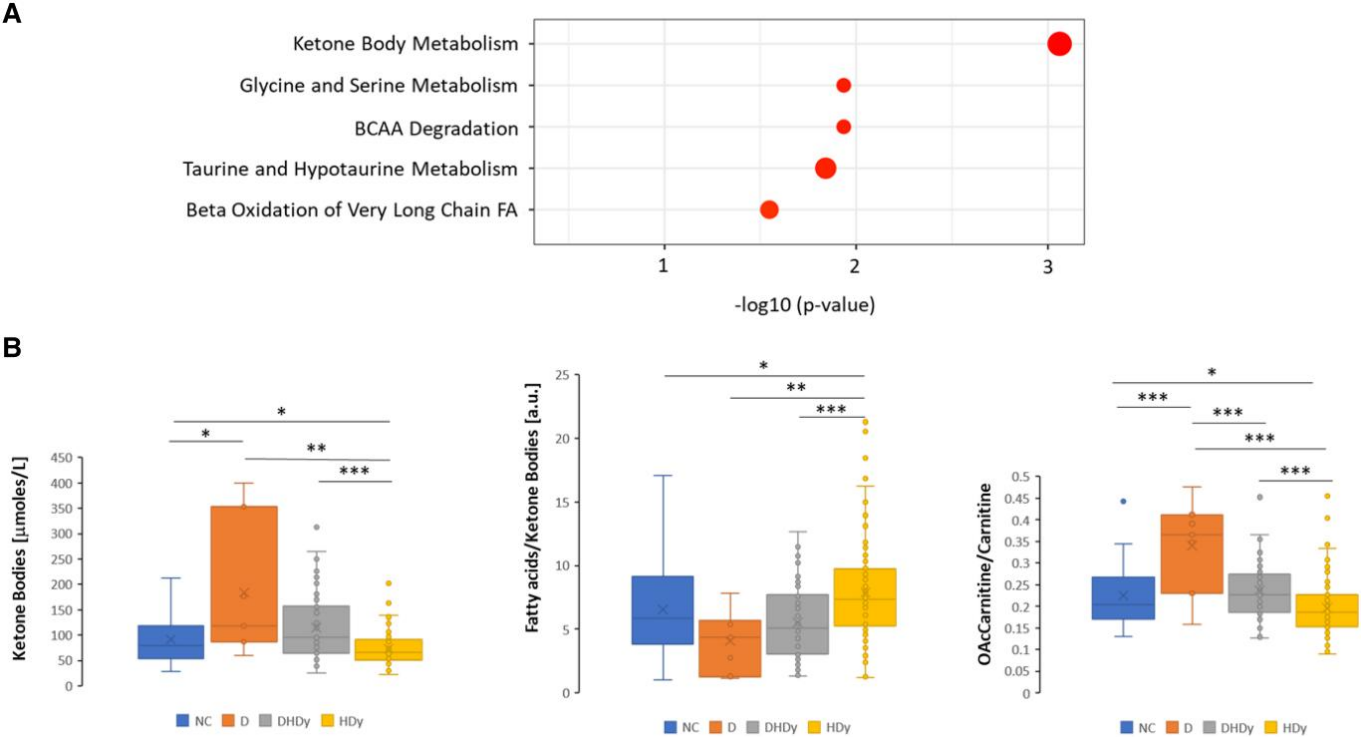
(A) Pathways that resulted regulated differently in diabetes compared to HDy. The dot size indicates the enrichment ratio from 5 to 20. (B) Selected metabolites and their circulating levels in subjects with NC, only D, DHDy, and HDy. The statistical significance between the different levels was calculated using the Mann–Whitney test, *** *P* < .001, ** *P* < .01, * *P* < –.05. Abbreviations: D, diabetes; DHDy, diabetes, hypertension, and dyslipidemia; HDy, hypertension and dyslipidemia; NC, no conditions.

Subsequently, the score contribution of metabolite associations that explain the different positions of HDy vs diabetes in Fig. 3 was calculated. Those metabolites showing the highest scores were used in an enrichment analysis by MetaboAnalyst, an online software tool (http://www.metaboanalyst.ca/) [21]. We found 5 pathways to be differently regulated in the 2 conditions. The most significant pathway was the metabolism of KBs, followed by glycine/serine metabolism, BCAA degradation, taurine and hypotaurine metabolism, and the β-oxidation of very long-chain FAs (Fig. 5A). We focused on 3 indicators related to the pathway showing the highest significance in the enrichment analysis: KB metabolism. Figure 5B displays the circulating levels of the sum of KBs, the ratio between FAs and KBs, and the ratio between O-acetylcarnitine and carnitine. The data belongs to the subjects with no condition, only diabetes, all 3 conditions, and hypertension and Dy.

Individuals with diabetes showed higher levels of circulating KBs than those without any of the 3 conditions. However, those with hypertension and Dy had lower levels of KBs compared to the group with no conditions. Consequently, individuals with all 3 conditions had KB concentrations close to those without any condition. The ratio between FAs and KBs showed an inverse behavior: values for diabetic subjects are lower compared to the control, while hypertensive and dyslipidemic individuals have higher values. Again, the comorbid situation showed values similar to those of the control group. Finally, the O-acetylcarnitine/carnitine ratio behaved similarly to the KB levels.

## Discussion

Our research explored the metabolic profiles of subjects at medium to high risk of CAD. Our objective was to study metabolic crosstalk among different disease states. Nearly two-thirds of the participants had multimorbidity situations, with a high prevalence of diabetes, hypertension, Dy, and some cases CKD. To accurately measure the level of polar metabolites and lipid families in plasma, we utilized NMR technology. Our laboratory has previously used this approach to examine the systemic response to Takotsubo syndrome [22].

As illustrated in Fig. 1, our results demonstrate the merits of the new representation used in our work. This approach combines the ability of multiple linear regression to extract those specific states that are associated with the metabolite concentrations with the intuitive representation of PCA that enables a comparison of diverse metabolic profiles. These plots allow us to compare diverse clinical conditions effectively. Additionally, the loading plot provides a simple yet informative representation of the relationship between metabolite concentrations and their impact on conditions, all within a single graph called a metabolic map.

We found an inverse correlation between female metabolic profiles and diabetes after accounting for age, treatment, and other diseases. This negative relationship was primarily due to the varying levels of specific metabolites such as BCAAs, KBs, and glycerolipids. High levels of BCAAs and KBs are strong indicators of diabetes [23], but women generally have lower BCAA concentrations [24]. Moreover, women typically exhibit lower fasting glucose and triglyceride levels than men [25], which are positively correlated with diabetes [26]. On the other hand, women have higher plasma glycine levels [24], which are inversely related to the risk of type 2 diabetes (T2D) [27].

Our research shows an association between BCAA and Dy, which aligns with a study conducted with a large Japanese group [28]. Interestingly, we discovered that high cholesterol or triglycerides did not have a substantial association with the condition in our study, likely due to the effects of statin treatment. Additionally, we observed that only specific metabolites, such as arachidonic acid and eicosapentaenoic acid levels and increased creatine phosphate, were linked to hypertension. These findings are consistent with prior research that has identified these factors as being associated with hypertension [29, 30].

A further observation from our metabolic maps is the similarity between the patterns of aging and CKD. The CKD profile, extracted from only 9 patients, includes the expected positive correlation with creatinine concentrations and confirms recent studies that reported an increase in blood levels of metabolites associated with kidney function and the urea cycle. These metabolites comprise citrulline [31-33], ornithine [31], urea [34], choline [32], myo-inositol [32], and succinate [35]. The observed increase in the circulating level of TMAO is worth mentioning, which is consistent with other studies [36, 37]. TMAO is being studied intensely as a potential biomarker and therapeutic target for CKD [38]. Its level is associated with the occurrence and prognosis of CKD, and it can be a possible risk factor for the development of CKD [39]. We also found that some of these metabolites were elevated with aging, suggesting a connection between kidney function and its decline throughout life. Although the increase in circulating urea with age was known [40], it was only recently observed that the urea cycle and ammonia recycling pathways are altered with aging [41].

A second common feature between CKD and aging profiles is the observed decrease in global AA levels, particularly for CKD of EAAs, asparagine, and methionine. It is known that the overall amount of circulating AAs drops as people age, with a significant decrease in EAAs [24]. Asparagine and methionine also diminish with age [24], together with histidine, serine, and threonine [42]. Although less is known about the relationship between circulating AAs and CKD, patients dependent on chronic hemodialysis are prone to malnutrition, and AAs can also be lost during hemodialysis [43].

Our cohort's Phe/Tyr ratio is positively associated with age and CKD. This ratio has been observed to increase in patients with CKD for the last 50 years [44-46], confirming our findings. There is evidence of impaired phenylalanine hydroxylation, removal, and decreased tyrosine synthesis in CKD patients [47]. The same rise in this ratio was also related to age and attributed to chronic low-grade inflammation [48]. During inflammation, enzymes such as GTP-CH1 and IDO are stimulated in immune cells like monocytes/macrophages and dendritic cells. GTP-CH1 produces tetrahydrobiopterin tetrahydrobiopterin, a crucial enzyme cofactor, including phenylalanine-hydroxylase, which converts phenylalanine to tyrosine. However, reactive oxygen species can destroy tetrahydrobiopterin during inflammation, impairing the enzymatic reaction catalyzed by phenylalanine-hydroxylase. This can decrease the conversion of phenylalanine to tyrosine and affect the body's production of biogenic amines and neurotransmitters [48]. Considering these points, it is possible that chronic inflammation, present in CKD and aging, is the common source for the observed similar change in the Phe/Tyr ratio.

Although our study included patients with various comorbidities, we could accurately and selectively extract their distinct metabolic profiles thanks to the multiple logistic regression results, even though some conditions are represented by only a few subjects, as in the case of CKD. By combining these results with the strength of PCA, we could quickly compare the metabolic profiles of various conditions. We used this analysis to validate our results by comparing them with metabolic profiles obtained in previous studies that focused on each condition individually to evaluate the efficiency of this approach to extract the principal biomarkers from a mixed population.

In fact, as a next step, we explored a more ambitious scenario to investigate potential compensatory metabolic effects in comorbid situations. Our focus was on the most prevalent combinations of diabetes, hypertension, and Dy within our study cohort, a group that presents significant clinical challenges. Hypertension is a critical risk factor for cardiovascular disease and affects up to 75% of T2D patients globally [49]. Controlling blood pressure effectively is essential to minimize complications in these patients, but it remains a challenging goal [50]. Dyslipidemia is a common comorbidity in T2D, with limited efficacy of statins in lowering triglycerides and lipoprotein(a) levels [51]. There is an urgent need for more effective treatments to manage both hypertension and Dy in T2D patients [52].

Considering the therapy challenges for individuals presenting all these 3 conditions, we concentrated on the metabolic compensation effects to search for possible causes based on systemic metabolism. The distinct positions of the HDy and diabetes states on the metabolic map in Fig. 3 already suggest that there are processes that show opposing alterations. Enrichment analysis of metabolites showing an inverse correlation between HDy and diabetes revealed that the primary distinction between these 2 states is the interplay between KB metabolism and FA β-oxidation. Ke et al's research established a connection between hypertension and incomplete FA breakdown, marked by the buildup of long-chain acyl CoAs, acylcarnitines, and diglycerides, indicative of incomplete FA β-oxidation [53]. Since KBs are produced in liver mitochondria during this process, this mechanism could account for the lower KB levels and increased FA/KB ratios found in individuals with hypertension and Dy, as opposed to those without these conditions. Conversely, diabetes often leads to increased blood KBs due to low insulin and high counterregulatory hormone levels [54]. Intriguingly, this contradictory pattern is mirrored in the heart muscle, where FA oxidation varies with heart failure type: it is increased in diabetes-induced heart failure but decreased in heart failure due to hypertension or ischemia [55].

The ratio of oAcC to Car, closely related to KB and FA metabolism, shows an inverse variation between diabetes and HDy, contributing to their opposing position in the metabolic map. Car and oAcC are vital in lipid oxidation and glucose metabolism, aiding FA transport into mitochondria, activating the glycolytic pathway, and stimulating the pyruvate dehydrogenase complex. Intravenous Car treatment enhancing insulin sensitivity in T2D aligns with our observation of a high oAcC/Car ratio in diabetes [56], implying a relative Car deficiency. Conversely, oAcC administration in a pilot hypertension study reduced systolic blood pressure in nondiabetic individuals [57]. To strengthen this point, a recent review pooling the results of 22 randomized controlled trials showed that Car supplementation in adults does not significantly affect blood pressure [58]. Our results showed a lower value of the OAcC/Car ratio in individuals with hypertension and Dy with respect to the control, and the external supply of OAcC would follow the rationale of regularizing this ratio.

The contrasting oAcC/Car ratio trends between diabetes and HDy reflect the differential use of Car and oAcC in their treatments. However, oAcC's impact on blood pressure, cholesterol, triglycerides, and glucose levels was not significant in patients on statin therapy with hypertension, diabetes, and Dy [52]. It is possible that the opposing effects of hypertension/Dy and diabetes on the breakdown of FAs through β-oxidation may reduce its importance as a target for therapy.

Our study provides valuable insights into the metabolic crosstalk among comorbidities, which could lead to more targeted treatments and improved therapeutic outcomes. A more comprehensive understanding of metabolic interplay in comorbid states can be achieved by including information about the severity and duration of the diseases. This point demands further examination, aiming to broaden the scope of both the quantity and quality of patient data obtained at enrolment. Although the limited number of subjects analyzed makes it difficult to draw definitive conclusions, our findings suggest a promising direction for future research.

## Data Availability

The raw data can be obtained at the request of the corresponding author.
